# Some Points Concerning Vaccination

**Published:** 1882-04

**Authors:** 


### SOME POINTS CONCERNING VACCINATION.

At a recent meeting of the St. Louis Med.-Chir. Society, Dr. W. A. Hardaway made the following remarks :

" Whenever an epidemic of small-pox occurs, it is the habit of many physicians to refer the prevalence of the disease to the neglect of vaccination by the laity. Now, while this is to a certain extent true, I submit that, as a profession, we are not altogether blameless in the matter. Therefore, I think it important to lay great stress on certain facts connected with vaccination, to wit : 1. As regards virus, I think that we are all well assured that bovine virus is as efficient in its protective influence, if not more so, as the best selected humanized stock, and as to its causing greater local disturbance my own experience quite negatives the idea. But it must always be borne in mind that much carelessness may be displayed in the selection of calf lymph, particularly when there is a great commercial demand for it, and that our spurious results are sometimes due to this fact. Other things being equal, however, the great advantage rests with the bovine virus, for the very obvious reason that by its use we set at rest popular prejudices in regard to the transmission of disease.

" 2. My second proposition relates to the number of vesicles which the practitioner should endeavor to produce. Dr. Seaton has well said that, in order that persons should have the fullest protection from variola which vaccination is capable of affording, it is necessary that the latter should not only be perfect in character, but also sufficient in amount. Persons whose vaccination has produced but one genuine vesicle are, as a rule, much less protected than are those who have had two, and so on ; and the protection given by four genuine vesicles is almost absolute. Mann's well-known table, which shows the influence exerted on small-pox by the number, as well as the character, of the vaccine marks, proves this position very thoroughly. I believe that, where the method of scarification is employed, the usual one in this country, there should be at least three large abrasions made, in order to be quite sure that we have sufficiently infected the system.

"3. The third point I wish to make, and one that is largely neglected, relates to the inspection of patients after vaccination has been performed. The idea of a 'take' is a very vague and general one in the popular mind, and by allowing medically uneducated people to be the judges of a successful inoculation, we needlessly expose them to risk of small-pox, and bring great discredit upon vaccination. I think that we should urge upon the profession the vital necessity of inspection, particularly from the eighth to the twelfth days, in order that we may assure ourselves of successful results. During my term of service as small-pox inspector I saw numerous cases of variola in persons with bad marks, or no marks, who had been confidently relying upon their immunity, because in their private judgments they had undergone efficient vaccination.

"4. I think that it is part of our duty to educate the people up to the idea of systematic revaccination. The necessity for revaccination depends, perhaps, upon the goodness of the primary result; but even the most conservative are agreed as to the utility of its repetition. Seaton urges that even the most thoroughly vaccinated should again undergo the operation. I think that numerous facts point to the conclusion that the immunity granted by vaccinal inoculation is not for so long a period as was once thought, and I can see no reason why a practically harmless operation should not be performed at stated intervals. I believe it to be a good rule to revaccinate everybody, regardless of the length of time which has elapsed after previous vaccination, whenever an epidemic is threatened—certainly not to wait until it is upon us."—*St. Louis Courier of Medicine.*

